# Nacre-like Anisotropic Multifunctional Aramid Nanofiber Composites for Electromagnetic Interference Shielding, Thermal Management, and Strain Sensing

**DOI:** 10.3390/molecules29174000

**Published:** 2024-08-23

**Authors:** Jin Dong, Jing Lin, Hebai Zhang, Jun Wang, Ye Li, Kelin Pan, Haichen Zhang, Dechao Hu

**Affiliations:** 1 School of Applied Physics and Materials, Wuyi University, Jiangmen 529020, China13848610003@163.com (H.Z.);; 2 School of Materials and Energy, Foshan University, Foshan 528000, China

**Keywords:** aramid nanofibers, composite films, electromagnetic interference shielding, thermal conductivity, strain sensing

## Abstract

Developing multifunctional flexible composites with high-performance electromagnetic interference (EMI) shielding, thermal management, and sensing capacity is urgently required but challenging for next-generation smart electronic devices. Herein, novel nacre-like aramid nanofibers (ANFs)-based composite films with an anisotropic layered microstructure were prepared via vacuum-assisted filtration and hot-pressing. The formed 3D conductive skeleton enabled fast electron and phonon transport pathways in the composite films. As a result, the composite films showed a high electrical conductivity of 71.53 S/cm and an outstanding thermal conductivity of 6.4 W/m·K when the mass ratio of ANFs to MXene/AgNWs was 10:8. The excellent electrical properties and multi-layered structure endowed the composite films with superior EMI shielding performance and remarkable Joule heating performance, with a surface temperature of 78.3 °C at a voltage of 2.5 V. Additionally, it was found that the composite films also exhibited excellent mechanical properties and outstanding flame resistance. Moreover, the composite films could be further designed as strain sensors, which show great promise in monitoring real-time signals for human motion. These satisfactory results may open up a new opportunity for EMI shielding, thermal management, and sensing applications in wearable electronic devices.

## 1. Introduction

With the rapid progress of highly integrated electronic telecommunication technology, the inevitable electromagnetic interference (EMI) radiation pollution and undesirable heat accumulation have emerged as intractable issues in recent years [1,2,3,4]. These issues relating to electronic devices will severely reduce their working performance, shorten their life span, and even harm human health. Meanwhile, the flourishing growth of smart wearable technology poses more requirements of flexibility and sensing performance for electronic devices [5,6]. Therefore, developing multifunctional flexible materials with high-performance EMI shielding, thermal management, and sensing capability is urgently required for next-generation smart electronics.

Aramid nanofibers (ANFs), well known as nanoscale Kevlar fibers, are composed of aligned poly(paraphenylene terephthalamide) (PPTA) chains, which can be derived from the deprotonation of macroscopic Kevlar fabrics or synthesized through a bottom–up approach [7,8,9]. Owing to their high anisotropy ratio and the strong interactions between PPTA chains, such as hydrogen bonding, π–π stacking, and van der Waals forces, ANFs exhibit exceptional mechanical properties and thermal resistance, serving as promising candidates for reinforcing phases and high-performance polymer substrates in flexible electronic materials [10,11,12]. However, the intrinsic non-conductive and non-magnetic characteristics render ANFs unable to shield against EMI. Currently, various conductive nanomaterials, including graphene, carbon nanotubes, Ag nanowires (AgNWs), and carbon dots, have been widely employed to enhance the conductivity of ANFs matrices [13,14,15]. Among these fillers, two-dimensional MXene (Ti_3_C_2_T_x_) has emerged as a popular material for fabricating conductive composites due to its excellent metallic conductivity, abundant surface functional groups, and good mechanical properties [16,17,18]. Great efforts have been devoted to improving the EMI shielding and thermal management capabilities of fibers with MXene. Additionally, the introduction of MXene can also endow fibers with various new functionalities, such as sensing physical deformations for monitoring human motion [19,20]. For instance, Li et al. successfully prepared wearable strain/pressure sensors with excellent sensing performance based on flexible and conductive MXene/cellulose nanocrystal (CNC)-coated thermoplastic polyurethane (TPU) nonwoven fabrics (NWFs) [21]. Similarly, Luo et al. reported a waterproof and breathable smart textile with a multicore shell structure via polydimethylsiloxane (PDMS) modifying MXene/elastomeric textiles, which exhibited excellent and durable photothermal and electrothermal conversion properties [22]. Although great progress has been made in improving the versatility of polymer composites by using MXene, several challenging issues remain. On the one hand, MXene nanosheets covering the gaps between fibers can reduce the conductive stability. On the other hand, achieving high electrical conductivity in polymer composites usually requires a large amount of MXene, which will cause poor interfacial interaction and thus reduce mechanical flexibility [23,24,25]. Consequently, the optimization of the conductive network in polymer matrices has attracted widespread attention [26,27,28,29]. Cheng et al. constructed a continuous thermal conductivity network by dip-coating magnetized nickel (Ni)/MXene (NiM) on a melamine foam (MF) template [30]. The synergistic effect of the magnetic Ni chain and MXene enables suitable thermal conductivity, high electrical conductivity, an excellent EMI shielding effect, and high latent heat storage capabilities. Zhao et al. prepared thin and flexible multi-layer alternating conductive gradient cellulose nanocomposite paper (CNF-MXene/AgNWs) using the alternating vacuum filtration process. The paper exhibited high mechanical strength, excellent EMI shielding performance, and outstanding thermal management performance [31]. The above studies fully demonstrate that constructing hybrid conductive networks with different structured fillers is a highly effective approach for achieving electron and phonon transport pathways in polymer matrices and enhancing their comprehensive performance.

Nature has imaginatively designed a variety of impressive and unique layered structures during the long-term processes of natural selection and evolution, providing inspiration for us in the manufacturing of anisotropic multifunctional composite films [32,33]. Herein, inspired by the highly oriented hierarchical architecture of natural nacre, nacre-like multifunctional ANFs/MXene/AgNW films with EMI shielding, thermal management, flame retardant, and strain-sensing capabilities were successfully prepared via vacuum-assisted filtration and hot-pressing. The formed three-dimensional (3D) anisotropic hybrid conductive networks between adjacent layered ANFs, AgNWs, and MXene facilitated a short electron transport path and fast phonon transport channel, thus resulting in high electron conductivity and exceptional in-plane thermal conductivity. As a result, owing to the strong internal multiple scattering of electromagnetic waves and reduced phonon scattering, the optimized films showed superior EMI shielding effectiveness (25 dB) and high thermal conductivity (6.4 W/m·K) when the mass ratio of ANFs to MXene/AgNWs was 10:8. Additionally, the compact and ordered structure endowed ANFs/MXene/AgNWs films with a high tensile strength of 83.86 MPa and an elongation at break of 8.41%. Meanwhile, the fabricated films also exhibited excellent Joule heating capabilities, outstanding nonflammability, and strain-sensing capabilities. Theoretically, these functions endow our designed films with great potential applications in wearable electronic devices. 

## 2. Results and Discussion

### 2.1. Preparation and Structural Characterization of ANFs/MXene/AgNWs Composite Films

**Figure 1:**
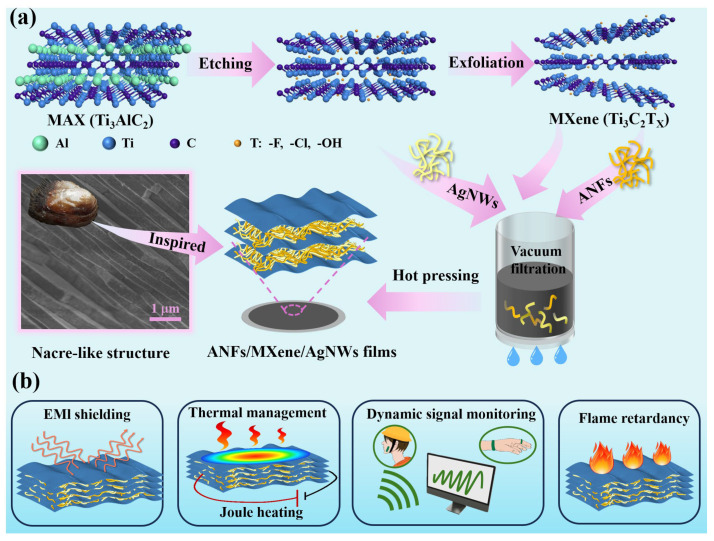
(**a**) The fabrication process of ANFs/MXene/AgNWs composite films. (**b**) A schematic diagram of the application of ANFs/MXene/AgNWs composite films in a wearable electronic device with excellent EMI shielding, thermal management, human motion monitoring, flame retardancy, and Joule heating abilities.

The preparation process of the ANFs/MXene/AgNWs composite films is depicted in Figure 1a and described in detail in the Materials and Methods. The hydrogen bond force between ANFs, AgNWs, and MXene facilitates the formation of highly stable and homogeneous dispersions. In addition, 1D structured fibers and nanowires can be well dispersed in 2D layered structured materials. Therefore, ANFs, MXene, and AgNWs can be intertwined to form nacre-like layered structure composite films via vacuum filtration. After hot-pressing, a dense 3D conductive network was constructed in the ANFs/MXene/AgNWs composites. Benefiting from the advantages of a hybrid conductive network, the prepared films can be applied in a wearable electronic device for EMI shielding, thermal management, human motion monitoring, flame retardancy, and Joule heating (Figure 1b).

**Figure 2:**
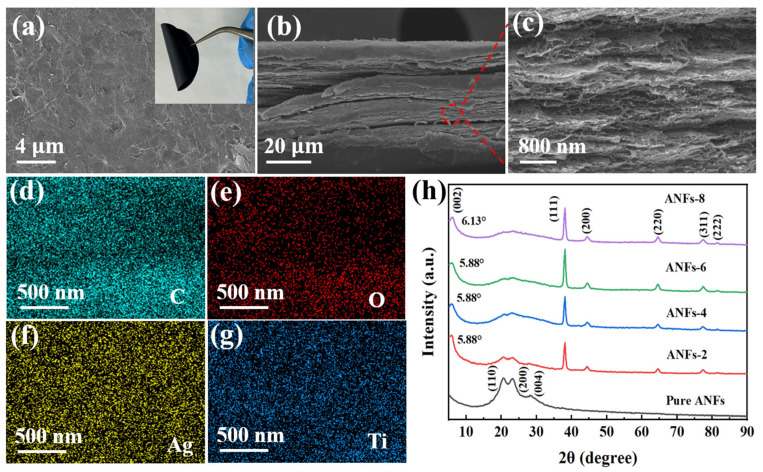
(**a**) Surface and (**b**,**c**) cross-section SEM images of ANFs-4 films; insert corresponds to optical photograph of ANFs-4 films with bending. (**d**–**g**) EDS mapping images of fractured surfaces of ANFs-4 films. (**h**) XRD patterns of pure ANFs and ANFs/MXene/AgNWs films.

As shown in Figure S1a, ANFs present a large aspect ratio with a radial size of ~10 nm and a length of several micrometers. Through the wet chemical method, Ti_3_AlC_2_ can be effectively exfoliated into a few layered Ti_3_C_2_T_x_ (MXene) nanosheets (Figure S1b). Additionally, AgNWs exhibit a small average diameter of ~30 nm (Figure S1c). Notably, the obtained ANFs, MXene and AgNWs dispersions show the typical Tyndall effect, indicating their colloidal characteristic and good uniformity. The mixing of ANFs, MXene, and AgNWs dispersions enables the uniform wrapping and twining of ANFs and AgNWs on MXene nanosheets with restrained self-aggregation, leading to a homogeneous and stable ANFs/MXene/AgNWs dispersion. As illustrated in Figure S2, the mixed composite films show almost the same deep black color on the upper and lower surfaces. Furthermore, good flexibility is observed in the films (Figure 2a). Figure 2a–c and Figure S2 show the surface and cross-sectional SEM images of the ANFs/MXene/AgNWs composite films. The surface SEM images reveal that the MXene nanosheets are densely stacked. Additionally, the fracture surface of the films possesses a dense and oriented layered structure, with MXene, ANFs and AgNWs being inserted in the composites, which is similar to the “brick-and-mortar” structure of nacre. The highly oriented hierarchical architecture endows the natural nacre with excellent mechanical properties. Inspired by a biomimetic structure, the fabricated filler network can prevent cracks from propagating to the whole film, and it is responsible for enhancing the mechanical properties. It is worth noting that the layered structure becomes more obvious and denser with increasing filler, and more filamentous materials can be observed on the fractured surface of the films (Figure S2). MXene acts as the skeleton of the layered structure. ANFs, as a “bonding agent”, can bridge the layered structures composed of MXene due to the formed hydrogen bond interactions. Additionally, 1D AgNWs are embedded into the whole film as conductive filler. In addition, the elemental distribution of representative C, O, Ti, and Ag suggests that ANFs, MXene, and AgNWs are uniformly dispersed in the films (Figure 2d–g). Additionally, the dense distribution of Ti elements indicates that the MXene nanosheets form a continuous network, further confirming the nacre-like layered structure of composite films [34,35]. Thus, the complementary integration of MXene and AgNWs builds a continuous and highly efficient 3D conductive network under the linkage of ANFs, resulting in effective electron transport and electrical conductivity [14]. 

Figure 2h shows the XRD patterns of pure ANFs films and uniformly mixed films with different filler contents. It is clear that the ANFs/MXene/AgNWs films inherit the physical characteristics of ANFs, MXene, and AgNWs. Additionally, the diffraction peak corresponding to the (002) crystal planes of MXene has a negative shift compared to pure MXene nanosheets, which is ascribed to the expansion in interlayer spacing through embedding ANFs [14,36,37]. Compared to ANFs-2, ANFs-4, and ANFs-6, one can see that the peaks of (002) crystal planes of MXene move from 5.88° to 6.13° for ANFs-8 films, indicating that the interlayer filling by ANFs and AgNWs has reached saturation, and the residual MXene nanosheets become dense, leading to the decreased interlayer spacing of MXene. In addition, pure ANFs present the typical diffraction peaks assigned to the (110), (200) and (004) crystal planes. The appearance of the (200) crystal face in pure ANFs demonstrates the regular arrangement of the molecular chain structure in ANFs and the abundant hydrogen bond interaction [38,39], while the intensity of the crystallization peak of ANFs is greatly reduced with increases in MXene and AgNWs. This indicates that the hydrogen bond in ANFs is broken and recombined with MXene, which results in the dissociated molecular chains of ANFs [40]. 

### 2.2. Mechanical Property, Electrical Conductivity and EMI Shielding Performance of ANFs/MXene/AgNWs Composite Films

**Figure 3:**
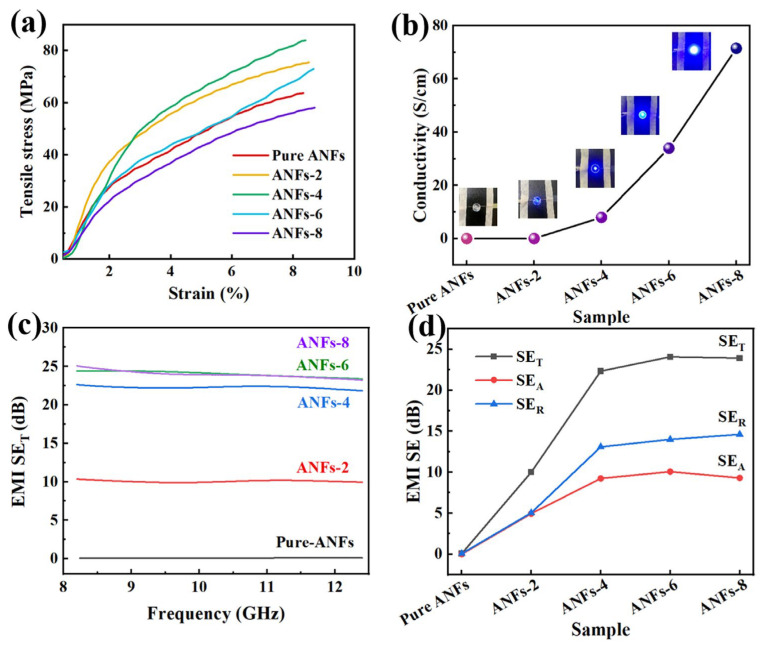
(**a**) Tensile stress–strain curves and (**b**) electrical conductivity of ANFs/MXene/AgNWs films. (**c**) Total EMI SE and (**d**) SE_R_, SE_T_, and SE_A_ of ANFs/MXene/AgNWs films.

The superior mechanical performance of composite films is the prerequisite for their wide application. In this work, a nacre-like layered structure was designed to optimize the mechanical properties of ANFs composite films. The typical tensile stress–strain curves of pure ANFs films and ANFs/MXene/AgNWs films with different filler loadings are shown in Figure 3a. It is clear that adding MXene and AgNWs can significantly increase the mechanical strength and slightly increase the fracture strain of the composite films. For example, the tensile strength of ANFs-4 films enhanced from 63.64 MPa to 83.86 MPa compared to pure ANFs films due to the strong interfacial interaction between MXene and ANFs/AgNWs (Figure S3). Meanwhile, a large loading of MXene/AgNWs will result in a decrease in mechanical properties. This is because the continuous increase in MXene may cause insufficient ANFs filling, resulting in weakened interlayer bonding and decreased mechanical properties. 

High electrical conductivity favors excellent EMI shielding performance. For ANFs/MXene/AgNWs composite films, the robust 3D conductive network of the multi-layer structures facilitates fast electron transport, contributing to high electrical conductivity. As shown in Figure 3b, the electrical conductivity of the composite films increases with increasing filler content. In particular, the electrical conductivity sharply increases from 7.98 to 33.98 S·cm^−1^ as the mass ratio of ANFs to MXene/AgNWs changes from 10:4 to 10:6 due to the formation of numerous junction points and more efficient MXene/AgNWs conductive networks. As a result, the optimized films have a high electrical conductivity of 71.53 S/cm with a mass ratio of 10:8 for ANFs/filler. The evolution of conductivity was further verified by examining the brightness of the LED lamp under different filler contents. Figure 3c,d display the EMI shielding performance of the nacre-like ANFs/MXene/AgNWs films in the X-band (8.2–12.4 GHz). As expected, the excellent EMI SE of the ANFs/MXene/AgNWs composite films is mainly attributed to the high electrical conductivity and their unique layered structure. The pristine ANFs had almost no capability to shield EM waves due to their insulation. The EMI SE of the ANFs-6 films reaches 25 dB, which is similar to that of ANFs-8 films. To study the shielding mechanism of ANFs/MXene/AgNWs films, data on the SE_T_, SE_A_ and SE_R_ are plotted in Figure 3d. It can be observed that the SE_R_ value of the composite films is obviously higher than the SE_A_ value, indicating that EM wave reflection is the main shielding mechanism [36]. It is noteworthy that the reflection of EM waves increases slowly after the mass ratio of ANFs and fillers increases to 10:4, which is ascribed to the perfect 3D network that formed inside the ANFs/MXene/AgNWs composite films. Moreover, the shielding efficiency also reflects the EM wave shielding capacity of the materials. As depicted in Figure S4, the composite films with ANFs/filler mass ratios of 10:6 and 10:8 are capable of blocking 99.99% of EM waves, suggesting that ANFs/MXene/AgNWs films possess excellent shielding properties [41]. The possible EMI shielding mechanism of the composite films is shown in Figure S5. When the electromagnetic wave irradiates the surface of the composite films, impedance mismatch occurs due to the large number of free electrons. Some of the incident electromagnetic wave is immediately reflected when it encounters the conductive layer, and the other part penetrates the films into the layered structure. The absorbed EM waves interact with the high density of carriers, resulting in many ohmic losses and the attenuation of the EM wave energy by the induced current. At the same time, the multi-layer structure will increase the propagation distance of the electromagnetic waves, and multiple internal reflections between layers also promote the absorption of EM waves. In addition, the local defects of MXene with abundant functional groups (-O, -OH, and -F) can lead to an asymmetric charge density distribution, thus forming local dipoles, resulting in the relaxation of polarization and an enhanced overall shielding effect [37,42,43]. The above mechanisms act simultaneously and result in the excellent capacity to attenuate and dissipate incident EM waves, thus inducing excellent EMI SE with a relatively high SE_A_ value.

### 2.3. Thermal Management and Joule Heating Performance of ANFs/MXene/AgNWs Composite Films

**Figure 4:**
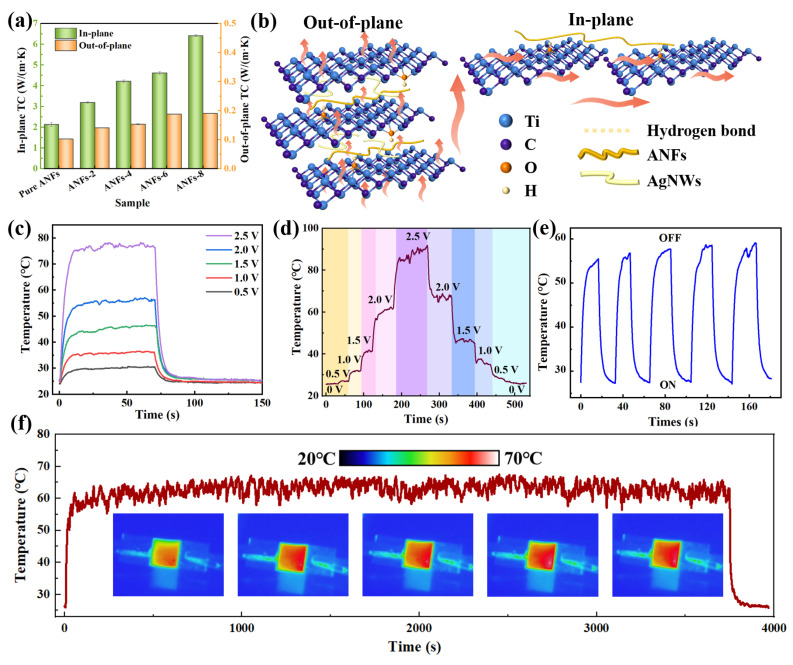
(**a**) Thermal conductivity and (**b**) schematic diagram of heat transfer model for ANFs/MXene/AgNWs composite films. (**c**,**d**) Surface temperature response of ANFs-8 films with different supplied voltages. (**e**) Surface temperature response of ANFs-8 films during five cycles at 2 V. (**f**) Long-term durability test of ANFs-8 films at 2 V.

Undesired heat generated during EMI shielding can significantly damage the performance of electronic devices. It is necessary to significantly enhance the thermal conductivity of EMI shielding materials. Figure 4a shows the in-plane and out-plane thermal conductivity (TC) values of pure ANFs and ANFs/MXene/AgNWs composite films. It was found that the TC displays an overall increasing tendency with the increasing filler content. Typically, ANFs-8 achieves an optimal in-plane TC of 6.4 W/(m·K), which is about six times higher than that of pure ANFs films. The fabricated composite films showed higher TC than many MXene/AgNWs-based composite films (Table S1). This is attributed to the fact that the parallel and close-contacting multi-layer structure of MXene can enhance planar heat conduction pathways, which contributes to high in-plane TC. Moreover, the out-plane TC of ANFs-8 also reached 0.19 W/(m·K) because ANFs and AgNWs can serve as the bridge between adjacent MXene to form a promising phonon and electron transportation channel (Figure 4b). 

Profiting from good electrical and thermal conductivity, ANFs/MXene/AgNWs composite films exhibit exceptional Joule heating capacity for thermal management applications, such as thermotherapy and self-heating smart garments. Figure S6 shows the surface temperature of the composite films and corresponding infrared thermal images, which vary depending on the voltages. As the voltage increases to 2.5 V, the maximum temperature of the composite films reaches 78.3 °C. Meanwhile, the obtained thermal infrared images show a uniform temperature distribution, which indicates that ANFs/MXene/AgNWs films can act as electric heaters. Figure 4d illustrates the surface temperature change in the composite films with different supplied voltages. During the process of electrical heating, the temperature rapidly reaches saturation as the input voltage increases (Figure 4c). The surface temperature of the films quickly exceeds a high temperature of 75 °C within 11 s at 2.5 V, which is attributed to the highly efficient and conductive 3D layered structure. Meanwhile, owing to the superior thermal conductivity of the ANFs-8 composite film, the surface temperature can drop to room temperature within 20 s as the voltage is turned off [14]. Figure 4d reveals that the surface temperatures of the composite films can be rapidly switched by applying different voltages, indicating their quick response to the change in voltage. Additionally, the saturation temperature is nearly proportional to the square of the input voltage, conforming to Joule’s law and demonstrating a highly controllable Joule heating capacity by tuning the supplied voltage (Figure S7) [44]. In addition, the obtained temperature change curves are almost identical when a voltage of 2 V is repeatedly supplied and removed, illustrating that the electrothermal properties of the composite films are highly stable and reliable (Figure 4e). Moreover, the ANFs/MXene/AgNWs films demonstrate a saturation temperature of 63 °C for 4000 s at an applied voltage of 2.0 V (Figure 4f), confirming their remarkable heating stability and durability under repeated, long-term working conditions. Ultimately, only a low voltage is required to achieve an excellent electrothermal effect, ensuring human safety in practical use and suggesting promising thermotherapy and self-heating smart garment applications. 

### 2.4. Thermal Stability and Flame Retardancy of ANFs/MXene/AgNWs Composite Films

**Figure 5:**
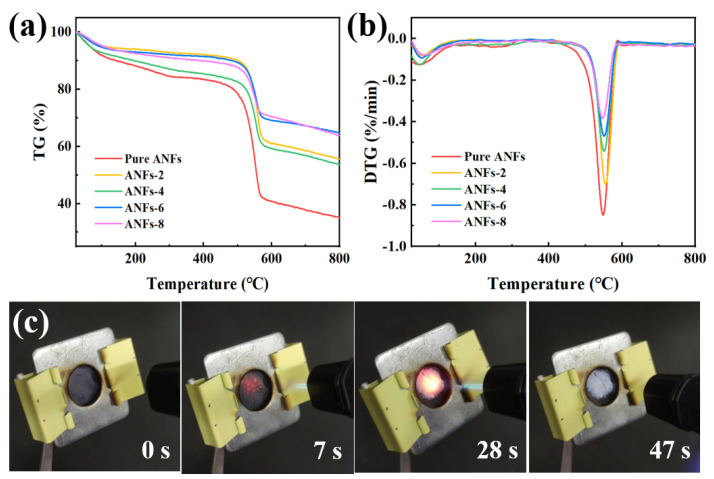
(**a**) TGA and (**b**) DTG curves of pure ANFs and ANFs/MXene/AgNWs composite films. (**c**) Digital images of burning behaviors of ANFs/MXene/AgNWs composite films.

The thermal stability of composite films in high-temperature environments is of paramount importance for their practical application [45,46,47]. The thermal decomposition behavior of pure ANFs films and composite films was investigated using TGA and derivative thermogravimetry (DTG). As depicted in Figure 5a, all of the samples display similar three-step weight loss from 100 °C to 800 °C. For pure ANFs films, the weight loss occurring at 350 °C is due to the evaporation of absorbed water and hydroxyl functional groups. The degradation of ANFs after 350 °C is attributed to the breakdown of the polymer backbone, leaving about 35% char residues at 800 °C. After adding MXene/AgNWs, the composite films show low decomposition rates in the range of 100–600 °C. Additionally, the weight loss of composite films decreased with an increase in the filler content, benefiting from the excellent thermal stability of MXene and AgNWs. Notably, the DTG curves reveal a significantly reduced peak weight loss rate with an increase in filler (Figure 5b). The decomposition temperature of ANFs undergoes an obvious increase with an increasing amount of filler. Typically, the T_10%_ value of ANFs-6 composite films increases from 143 to 485 °C compared to pure ANFs films due to the high thermal stability of the filler as well as the strong interfacial interactions between MXene and ANFs/AgNWs. Meanwhile, the residual mass of ANFs-6 composite films at 800 °C increases from 35.03% to 64.76% in comparison with pure ANFs films (Table S2). This is mainly attributed to the barrier effect caused by multi-layered MXene nanosheets, which hinders thermal diffusion and the transport of volatile species, simultaneously providing additional heat dissipation pathways [48,49]. 

To further evaluate the flame resistance of ANFs/MXene/AgNWs composite films, their burning behaviors were studied, as detailed in Figure 5c,d. Pure ANFs films are ignited immediately upon contacting the flame (within 1 s), showing complete incineration by 3 s (Figure S8). In contrast, the ANFs/MXene/AgNWs composite films retained their original shape and did not ignite after being exposed to the flame for 7 s (Figure 5c). A modest amount of white smoke was generated while burning, and white residual material gradually appeared under a prolonged flame of 47 s, indicating the oxidation and degradation of MXene to form TiO_2_. Furthermore, the flame would quickly be extinguished after removing the heat source, demonstrating excellent flame retardance and self-extinguishing properties [50].

### 2.5. Sensing Performance and Application of ANFs/MXene/AgNWs Composite Films

**Figure 6:**
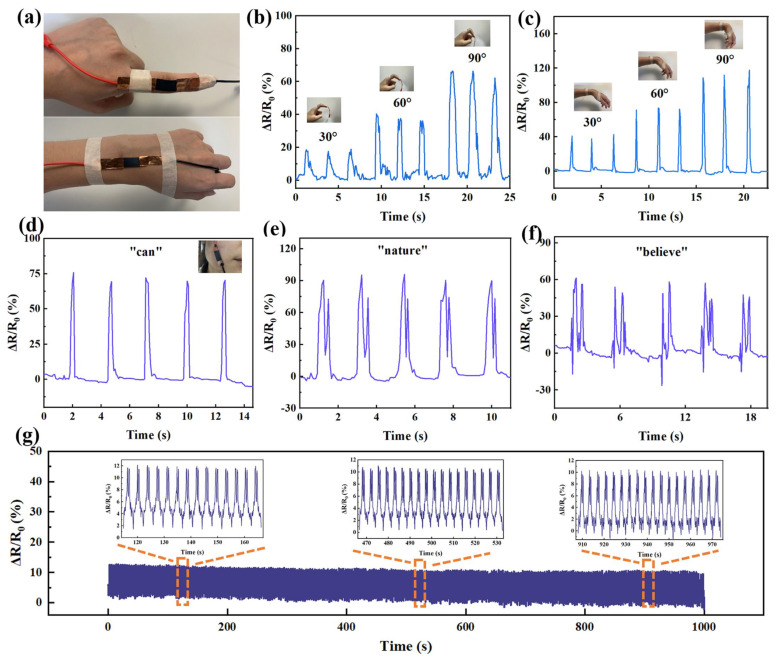
(**a**) A diagram of the sensor attached to the finger and wrist. (**b**,**c**) The resistance response of the sensor while monitoring the bending of the finger and wrist. (**d**–**f**) The resistance response of the sensor when monitoring the pronunciation of the words can, nature, and believe. (**g**) The resistance response of the sensor over 1000 s cyclic tests under 10° bending.

The internal structure of the composite films will be deformed to different degrees when stimulated by external forces. In this study, the as-prepared ANFs/MXene/AgNWs films could be fabricated as strain sensors. ΔR/R_0_ was used to represent the relative resistance changes during the bending process, where ΔR refers to the resistance change before and after bending, and R_0_ is the original resistance. Fabricated sensors attached to the finger and wrist were employed to monitor motion (Figure 6a). A fast and stable resistance response was observed when the finger was bent from 30° to 90° due to the slippage of the layered structure (Figure 6b). Similarly, the wrist bending angles can be judged based on the resistance change (Figure 6c). Moreover, the sensor attached to the cheek can recognize the pronunciations of different words, such as can, nature, and believe (Figure 6d–f). Obviously, single, double, and multi-syllable words show different peak characteristics of ΔR/R_0_. In addition, service life is also an important indicator of the sensor’s performance. As shown in Figure 6g, ΔR/R_0_ remains almost stable during the cycling test for 1000 s under 10° bending, demonstrating the long-time durability as a wearable electronic device. In practical applications, the movement statuses of firefighters or various special staff who need to act alone can be monitored using the designed sensor in real time or remotely sensed through a human–computer interaction system, which is conducive to timely rescue actions.

## 3. Materials and Methods

### 3.1. Materials 

Dispersed ANFs (0.6 wt.%) were purchased from Houpu Fiber Technology Co., Ltd (Kunshan, China). Titanium aluminum carbide powder (Ti_3_AlC_2_, 400 mesh) was supplied by Xiyan New Material Technology Co., Ltd (Heze, China). Lithium fluoride (LiF, AR, 99%) was purchased from Adamas. Hydrochloric acid (HCl, ~38%) and NaOH (AR, 99.5%) were obtained from Chemical Reagent Co., Ltd (Guangzhou, China). Dispersed AgNWs (5 mg/mL) were acquired from Leadernano Technology Co., Ltd (Jining, China). 

### 3.2. Synthesis of MXene Nanosheets 

MXene (Ti_3_C_2_T_x_) was prepared following the method described in our previous study [34]. First, 4.8 g of LiF was dispersed in 60 mL of 9 M HCl solution; 3 g of Ti_3_AlC_2_ powder was then slowly added and stirred for 24 h. Subsequently, the suspension was centrifuged several times until the pH of the supernatant was over 5. The obtained sediment was ultrasonically dispersed in water and then centrifuged for 1 h. Finally, the dark green supernatant was filtrated to obtain MXene.

### 3.3. Fabrication of the ANFs/MXene/AgNWs Composite Films 

An amount of 10 g of ANFs was dispersed in water and stirred using a high-speed mixer to form a homogeneous and stable ANFs dispersion. To determine the effect of the hybrid filler on the comprehensive properties of the ANFs composites, a different content of hybrid filler was emphasized. Thus, the definite mass ratio of MXene to AgNWs (1:1) was determined to better study the role of hybrid filler in ANFs composites. MXene and AgNWs were added to the above dispersion and stirred with a magnetic mixer for 1 h (the mass ratios of ANFs to MXene/AgNWs were 10:2, 10:4, 10:6, and 10:8 and the mass ratio of MXene to AgNWs was 1:1). Then, the solution was filtrated with a 1.2 μm filter membrane and hot-pressed at 60 °C for 3 h to obtain the ANFs/MXene/AgNWs films. The samples were denoted as pure ANFs, ANFs-2, ANFs-4, ANFs-6, and ANFs-8, respectively, which correspond to the mass ratios of 10:0, 10:2, 10:4, 10:6, and 10:8.

### 3.4. Characterization 

The morphologies of ANFs, MXene, and AgNWs were observed with a transmission electron microscope (TEM, JEM-F200, JEOL, Akishima, Japan). The surface and cross-section morphologies of the pure ANFs and ANFs/MXene/AgNWs films were obtained using a scanning electron microscope (SEM, FEI, NoVoTM nano SEM 430, Hillsboro, OR, USA). In addition, energy-dispersive X-ray spectrometry (EDS, Sigma 500 Oxford, Arlen, Germany) mapping was used to obtain the elemental distribution in the ANFs/MXene/AgNWs films. X-ray diffraction (XRD, X’Pert3Powder, PANalytical B.V., Eindhoven, The Netherlands) was used to observe the structure and composition of the samples. The electrical conductivity of the films was determined using a Hall effector (Ecopia HMS-5000, Seoul, Korea). Thermogravimetric analysis (TGA) using a thermal analyzer (TGA/DSC3+ LF/1100, METTLER TOLEDO, Zurich, Switzerland) was performed to verify the thermal stability of the samples at a temperature ranging from 25 to 800 °C and a heating rate of 10 °C/min under a nitrogen atmosphere. The real-time resistance of the ANFs/MXene/AgNWs films under various bending angles and pressures was recorded using a digital multimeter (Keithley 2450, Washington, DC, USA). The mechanical stability was investigated by using a tensile testing machine (TestometricX250-1, Rochdale, UK) with a stretch rate of 100 mm/min. The surface temperature of the composite films was recorded using an infrared thermal imager (InfReC R550, Avio, Japan). A laser-flash technique (LFA; NanoFlash 467, Netzsch, Bavaria, Germany) was used to measure the thermal conductivity of the ANFs/MXene/AgNWs films. The electromagnetic interference shielding effectiveness (EMI SE) of the ANFs/MXene/AgNWs films in the frequency range of 8.2–12.4 GHz (X-band) was performed using a vector network analyzer (Agilent PNA-N5234A, Washington, DC, USA). The reflection coefficient (R), absorption coefficient (A) and transmission coefficient (T) were calculated by measuring the scattering parameters (*S*_11_ and *S*_21_). The reflection shielding (SE_R_), absorption shielding (SE_A_), and total EMI shielding effectiveness (SE_T_) were obtained using the following formulas:

(1) $$R={\left|S_{11}\right|}^{2},\;A={\left|S_{21}\right|}^{2}$$

(2) A=1−R−T

(3) $${\mathrm{SE}}_{R}\left(\mathrm{dB}\right)=-10\mathrm{lg}\left(1-R\right)$$

(4) $${\mathrm{SE}}_{A}\left(\mathrm{dB}\right)=-10\mathrm{lg}\left[T/\left(1-R\right)\right]$$

(5) $${\mathrm{SE}}_{T}\left(\mathrm{dB}\right)=-10\mathrm{lgT}={\mathrm{SE}}_{R}+{\mathrm{SE}}_{A}$$

## 4. Conclusions

In summary, we successfully prepared nacre-like multifunctional flexible ANFs/MXene/AgNWs composite films with an anisotropic layered structure via vacuum-assisted filtration and hot-pressing. Benefiting from the hydrogen bonding interaction between ANFs, AgNWs, and MXene, the conductive fillers were uniformly dispersed in the NF matrix. The obtained composite films showed good flexibility, a high tensile strength of 83.86 MPa and an elongation at break of 8.41%. Meanwhile, ANFs and AgNWs acted as the bridge between the neighboring MXene nanosheets to form a robust 3D conductive network and thus provide an effective electron and phonon conduction pathway, which enables a high electrical conductivity of 71.53 S/cm and a superior thermal conductivity of 6.4 W/(m·K) when the mass ratio of ANFs to filler is 10:8. The composite films achieved outstanding EMI shielding performance due to their excellent electrical properties and multi-layer structure. Additionally, the composite films were found to have superior Joule heating properties, with a high surface temperature of 78.3 °C at a low voltage of 2.5 V, indicating their potential application in thermotherapy and self-heating smart garments. Furthermore, the composite films are fireproof and can maintain good structural integrity in the case of fire, thus expanding their application under harsh conditions. Additionally, composite films can be designed as strain sensors, monitoring real-time signals for human motion. The bio-inspired design of multifunctional ANFs composite films with superior EMI shielding, thermal management, flame retardancy, and sensing capabilities offers a new insight into expanding the widespread application of multifunctional composite films in wearable electronic devices.

## Data Availability

The data presented in this study are available upon request from the corresponding author.

## Supplementary Materials

The following supporting information can be downloaded at: https://www.mdpi.com/article/10.3390/molecules29174000/s1. Figure S1: TEM images of ANFs, MXene and AgNWs; Figure S2: Optical and SEM images of ANFs/MXene/AgNWs films; Figure S3: Tensile strength of ANFs films with different loading of MXene/AgNWs; Figure S4: Shielding efficiency of ANFs/MXene/AgNWs films; Figure S5: Schematic diagram of EMI shielding mechanism of ANFs/MXene/AgNWs films; Figure S6: Infrared images of ANFs-8 films at different voltages; Figure S7: Linear fitting of saturation temperature versus the U^2^ of the ANFs-8 films; Figure S8: Digital images of the burning behavior of pure ANFs. Table S1: EMI SE and TC of MXene/AgNWs-based composite films; Table S2: DTG results of pure ANFs and ANFs/MXene/AgNWs composite films. References [51,52,53,54,55,56,57] are cited in the Supplementary Materials.
