# Supplementary material for: Nacre-like Anisotropic Multifunctional Aramid Nanofiber Composites for Electromagnetic Interference Shielding, Thermal Management, and Strain Sensing
Source: Molecules. 2024 Aug 23;29(17):4000. doi: 10.3390/molecules29174000 (PMC11396044; doi:10.3390/molecules29174000)
Supplement: Supplementary file 1 [file molecules-29-04000-s001.zip › molecules-3162263-supplementary.pdf]

Supporting Information

Nacre-like anisotropic multifunctional aramid nanofiber composites for electromagnetic interference shielding, thermal management and strain sensing

Jin Dong [a], Jing Lin[a, *], Hebai Zhang [a], Jun Wang[a], Ye Li[a], Kelin Pan[a], Haichen Zhang[b], Dechao Hu[b, *]

[a] *School of Applied Physics and Materials, Wuyi University, Jiangmen 529020, China*

[b] *School of Materials and Energy, Foshan University, Foshan 528000, China*

*** Corresponding author**

* E-mail address: jinglin@wyu.edu.cn (J. Lin), msdchu@fosu.edu.cn (D. Hu).

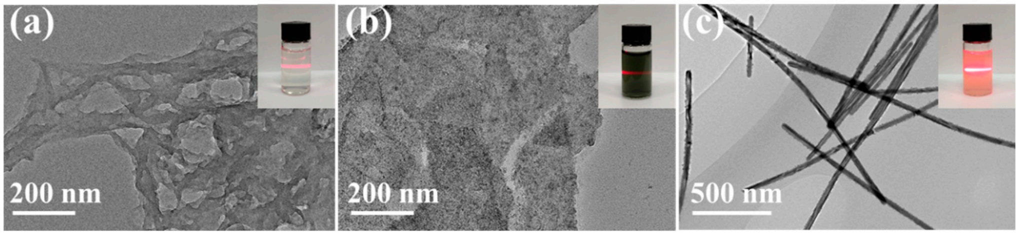

Figure S1. (a-c) TEM images of ANFs, MXene and AgNWs, respectively, the inserts correspond to their Tyndall effect.

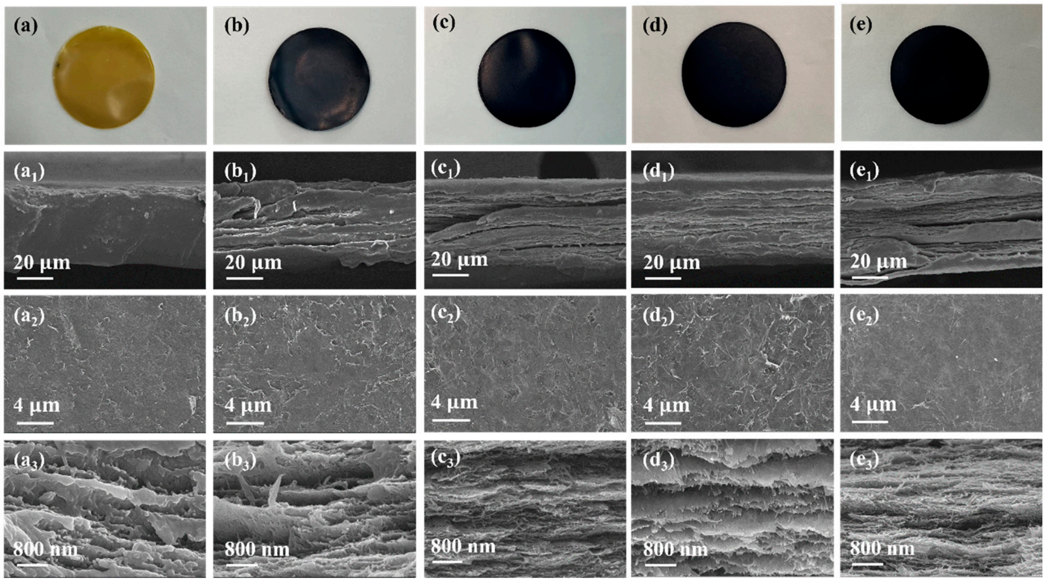

Figure S2. (a-e) Optical and SEM images of ANFs/MXene/AgNWs films (Pure ANFs, ANFs-2, ANFs-4, ANFs-6 and ANFs-8). (a_1-e_1) Cross-section SEM images. (a_2-e_2) Surface-section SEM images. (a_3-e_3) Enlarged cross-section SEM images.

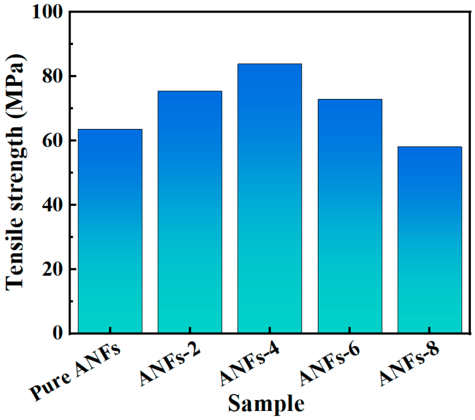

Figure S3. Tensile strength of ANFs films with different loading of MXene/AgNWs.

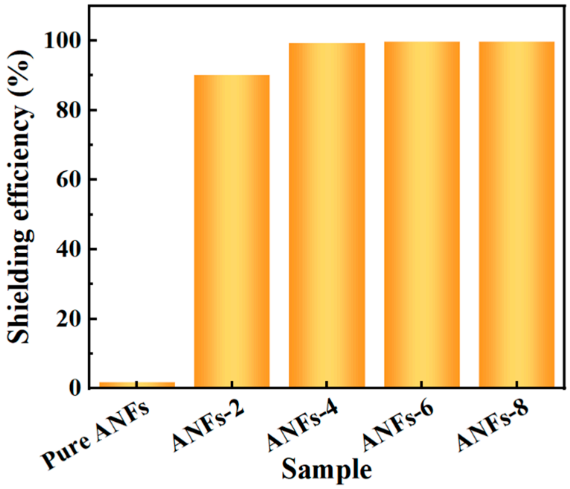

Figure S4. Shielding efficiency of ANFs/MXene/AgNWs films.

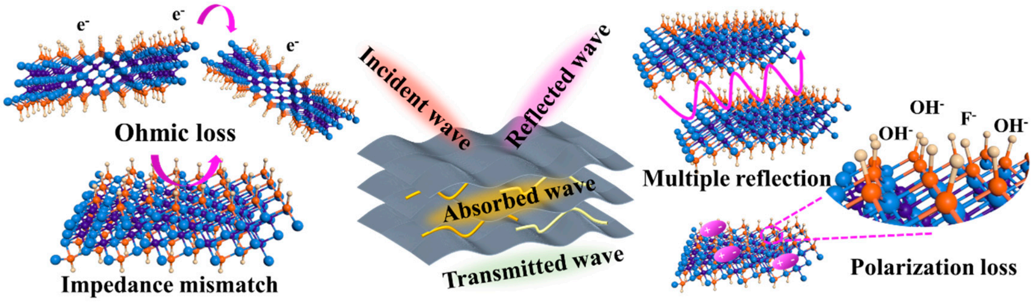

Figure S5. Schematic diagram of EMI shielding mechanism of ANFs/MXene/AgNWs films.

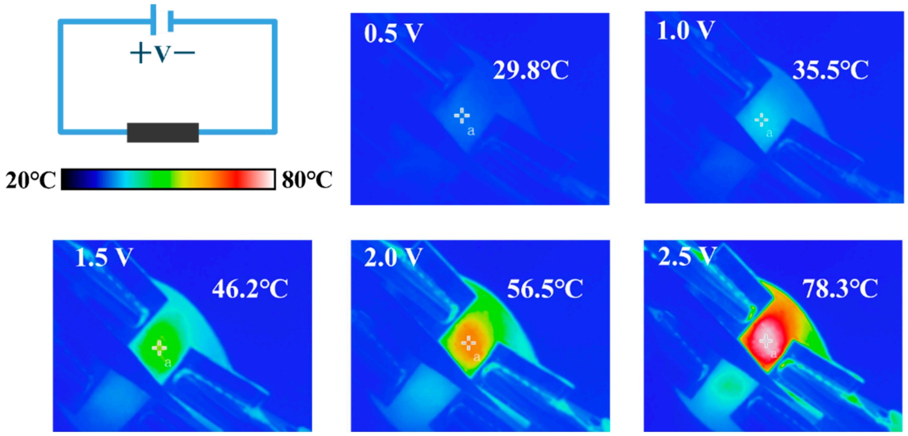

Figure 6. Infrared images of ANF-8 films at different voltages.

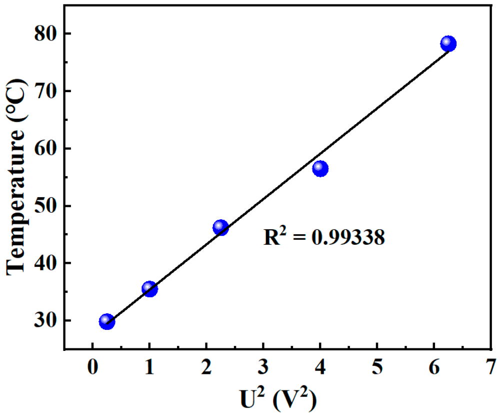

Figure 7. Linear fitting of saturation temperature versus U^2 of the ANF-8 films.

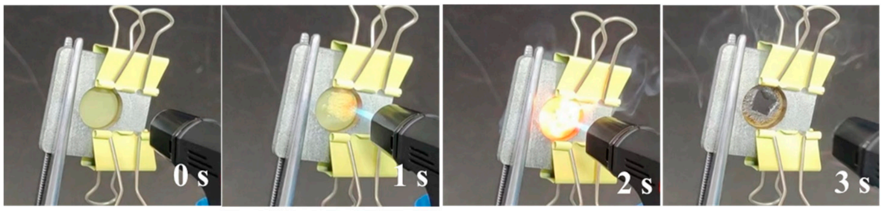

Figure 8. Digital images of burning behavior for pure ANFs.

Table S1. EMI SE and TC of MXene/AgNWs-based composite films.

Samples	EMI shielding effectiveness (dB)	Thermal conductivity (W/(m·K)	Reference
ANFs/MXene/AgNWs	-	0.49	[51]
PEG/MXene/AgNWs	-	0.64	[52]
PVB//MXene/AgNWs	30	-	[53]
PVA/MXene@AgNWs	32	3.72	[54]
PVDF/MXene/AgNWs	25.78	0.78	[55]
WPU/MXene/AgNWs	87.3	4.71	[56]
CNF/MXene-AgNWs@$CoFe_2O_4$	70.9	6.3	[57]
ANFs/MXene/AgNWs	25	6.4	This work

Table S2. TGA results of pure ANFs and ANFs/MXene/AgNWs composite films.

Samples	Residual weight at 800 °C (%)	$T_{10\%}$ (°C)
Pure ANFs	35.03	143.71
ANFs-2	55.68	500.65
ANFs-4	53.63	195.81
ANFs-6	64.76	485.56
ANFs-8	63.79	393.31

Reference

51. Tao, Y.; Mi, Y.; Gao, S.; Wang, G.; Bai, J.; Ma, S.; Wang, B. High-efficiency, thermal stable, and self-floating silver nanowires/Ti_3C_2Tx MXene/aramid nanofibers composite aerogel for photothermal water evaporation and antibacterial application. *Chem. Eng. J.* **2023**, *477*, 147276.
52. Ma, Y.; Zou, M.; Chen, W.; Luo, W.; Hu, X.; Xiao, S.; Luo, L.; Jiang, X.; Li, Q. A structured phase change material integrated by MXene/AgNWs modified dual-network and polyethylene glycol for energy storage and thermal management. *Appl. Energy* **2023**, *349*, 121658.
53. Han, X.; Feng, H.; Tian, W.; Zhang, K.; Zhang, L.; Wang, J.; Jiang, S. A Sandwich Structural Filter Paper–AgNWs/MXene Composite for Superior Electromagnetic Interference Shielding. *Polymers* **2024**, *16*, 760.
54. Li, M.; Sun, Y.; Feng, D.; Ruan, K.; Liu, X.; Gu, J. Thermally conductive polyvinyl alcohol composite films via introducing hetero-structured MXene@silver fillers. *Nano Res.* **2023**, *16*, 7820–7828.
55. Cheng, H.; Pan, Y.; Chen, Q.; Che, R.; Zheng, G.; Liu, C.; Shen, C.; Liu, X. Ultrathin flexible poly(vinylidene fluoride)/MXene/silver nanowire film with outstanding specific EMI shielding and high heat dissipation. *Adv. Compos. Hybrid Mater.* **2021**, *4*, 505–513.
56. Ye, X.-A.; Zhou, X.; Zeng, X.-Y.; Wang, G.-G. Conductive Composite Inks Comprised of Waterborne Polyurethane, Silver Nanosheets, and Heat-Treated MXene Nanosheets for Electromagnetic Shielding and Thermal Management. *ACS Appl. Nano Mater.* **2024**. https://doi.org/10.1021/acsanm.4c02902.
57. Guo, Z.; Ren, P.; Lu, Z.; Hui, K.; Yang, J.; Zhang, Z.; Chen, Z.; Jin, Y.; Ren,. F. Multifunctional $CoFe_2O_4$@MXene-AgNWs/Cellulose Nanofiber Composite Films with Asymmetric Layered Architecture for High-Efficiency Electromagnetic Interference Shielding and Remarkable Thermal Management Capability. *ACS Appl. Mater. Interfaces* **2022**, *14*, 41468–41480.
